# Influence of high-density lipoprotein cholesterol on coronary collateral formation in a population with significant coronary artery disease

**DOI:** 10.1186/1756-0500-6-105

**Published:** 2013-03-20

**Authors:** Po-Chao Hsu, Ho-Ming Su, Suh-Hang Juo, Hsueh-Wei Yen, Wen-Chol Voon, Wen-Ter Lai, Sheng-Hsiung Sheu, Tsung-Hsien Lin

**Affiliations:** 1 Department of Internal Medicine, Division of Cardiology, Kaohsiung Medical University, 100 Tzyou 1st Road, Kaohsiung, 80708, Taiwan, ROC; 2 Graduate Institute of Medicine, Kaohsiung Medical University, Kaohsiung, Taiwan; 3 Department of Medical Research, Kaohsiung Medical University Hospital, Kaohsiung, Taiwan; 4 Medical Genetics, Kaohsiung, Taiwan; 5 Center of Excellence for Environmental Medicine, Kaohsiung Medical University, Kaohsiung, Taiwan; 6 Department of Internal Medicine, Faculty of Medicine, Kaohsiung, Taiwan; 7 Department of Internal Medicine, Kaohsiung Municipal Hsiao-Kang Hospital, Kaohsiung, Taiwan

**Keywords:** Coronary artery disease, Coronary collateral circulation, High-density lipoprotein cholesterol

## Abstract

**Background:**

Coronary collateral circulation plays an important role in protecting myocardium from ischemia and reducing cardiovascular events. Low High-density lipoprotein cholesterol (HDL-C) level is a strong risk factor for coronary artery disease (CAD) and is associated with poor cardiovascular outcome. It was recently reported to be associated with poor coronary collateral development in Turkish population. Hence, we investigated the influence of HDL-C on coronary collateral formation in Chinese population.

**Methods:**

We evaluated 970 consecutive patients undergoing coronary angiography, and 501 patients with significant coronary artery disease (SCAD) were finally analyzed. The collateral scoring system developed by Rentrop was used to classify patient groups as those with poor or good collaterals.

**Results:**

The patients with poor collaterals had fewer diseased vessels (1.97 ± 0.84 vs 2.47 ± 0.68, p < 0.001) and lower diffuse score (2.65 ± 1.63 vs 3.76 ± 1.78, p < 0.001). There was no significant difference in HDL-C and other variables between good and poor collaterals. Multivariate analysis showed only number of diseased vessels (odd ratio 0.411, p < 0.001) was a significant predictor of poor collateral development.

**Conclusions:**

The extent of CAD severity but not HDL-C level was the most powerful predictor of coronary collateral formation in our Chinese population with SCAD.

## Background

Cardiovascular disease is one of the leading causes of morbidity and mortality in the world. Low high-density lipoprotein cholesterol (HDL-C) is an independent risk factor for coronary artery disease (CAD) and is associated with increased cardiovascular event [1-3].

The development of coronary collaterals is an adaptive response to chronic myoischemia and serves as a conduit bridging the significantly stenotic coronary vessels [4-6]. Collateral circulation can hence protect and preserve myocardium from episodes of ischemia, enhance residual myocardial contractility, and reduce angina symptoms and cardiovascular events [7-9]. However, there is inter-individual difference of coronary collateral formation and the mechanisms for different individual ability to develop collateral circulation are still not well understood.

Furthermore, HDL-C was also recently reported to be associated with poor coronary collateral development in a Turkish population [10]. Hence, we wanted to investigate the influence of HDL-C on coronary collateral formation in a Chinese population with significant CAD (SCAD).

## Methods

### Study subjects

From February 2002 to March 2008, we evaluated 970 consecutive patients who had been scheduled to undergo diagnostic coronary angiography at Kaohsiung Medical University Hospital in Taiwan. We excluded patients with coronary artery lumen diameter stenosis < 70%, history of coronary artery bypass surgery (CABG), history of percutaneous coronary intervention (PCI), or those who had inadequate angiograms for collateral evaluation. Finally, 501 patients with significant CAD were enrolled in this study. Other analyzed demographic and baseline data included gender, age, and any history of the following: diabetes mellitus, hypertension, hypercholesterolemia, body mass index, cigarette smoking, and medications. The research protocol was approved and registered by the ethics committee (Kaohsiung Medical University Hospital- Institutional Review Board) at our institution, and informed consent was obtained from all patients.

### Coronary angiography and collateral scoring

The coronary artery angiography films were reviewed by 2 experienced cardiologists blinded to the clinical and demographic data for all patients. Any differences in interpretation were resolved by a third reviewer who was blinded to the readings of the first two reviewers. Coronary artery stenosis was determined by quantitative coronary angiography. The recorded data also included the number of diseased vessels, the vessel to which the collaterals were connected, and the grade of coronary collateral circulation. Vessels exhibiting a 70% or greater reduction in lumen diameter were classified as SCAD. In subjects with more than one SCAD vessel, the vessel with the highest collateral grade was chosen for analysis. The collateral scoring system developed by Rentrop and Cohen was used [11]. Grades of collateral filling from the contralateral vessel were: 0 = none; 1 = filling of side branches of the artery to be dilated via collateral channels without visualization of the epicardial segment; 2 = partial filling of the epicardial segment via collateral channels; 3 = complete filling of the epicardial segment of the artery being dilated via collateral channels. In subjects with more than one collateral vessel supplying the distal aspect of the diseased artery, the highest collateral grade was recorded. Patients were then classified according to their collateral grades as either poor (grade 0 or grade 1 collateral circulation) or good (grade 2 or grade 3 collateral circulation). The coronary artery angiography films were reviewed by two experienced cardiologists blinded to the clinical and demographic data for all patients. Any differences in interpretation were resolved by a third reviewer who was blinded to the readings of the first two reviewers. The two readers obtained a 96% agreement in the collateral classifications.

### CAD severity scoring by coronary angiography

We evaluated the extent of coronary artery atherosclerosis by a “diffuse score” (DS) developed by Negri et al. and modified by Birnie et al. [12,13]. In brief, the coronary circulation is divided into 15 segments, and eight of them are classified as first-order segments: proximal and middle right coronary arteries, left main coronary artery, proximal, middle, and distal left anterior descending, and proximal and distal circumflex. There are seven second-order segments: distal right coronary artery, posterior descending branch, obtuse marginal branch, postero-lateral branch of circumflex, and the first two diagonal branches of the left anterior descending artery. The first-order segments receive a score of 1 and the second order segments scored 0.5. The overall DS is the sum of the individual segment scores and the maximum score is 11.5.

### Statistical analysis

All data were expressed as means ± standard deviation. Student unpaired *t*-test was used to compare continuous variables between the two groups. Chi-square test was used to compare categorical data. Subsequently, age, sex, significantly correlated variables in the univariate analysis or relevant variables were further analyzed by logistic regression analysis. All p values were two-sided with a significance level of p < .05. The Statistical Package for the Social Sciences 11.0 for Windows (SPSS Inc., Chicago, IL) was used for statistical analysis.

## Results

### Clinical characteristics

Of the 970 subjects initially enrolled, 469 patients were excluded for the following reasons: coronary artery lumen diameter stenosis < 70%, history of CABG or PCI, or inadequate angiograms for collateral evaluation. The final study population was 501 subjects (396 male, 105 female; average age, 62.5 ± 12.6 years). For SCAD, 134 patients (26.7%) were one-vessel disease, 153 patients (30.5%) were two-vessel disease, and 214 patients (42.7%) were three-vessel disease.

### Coronary collaterals

The two readers obtained a 96% agreement in the collateral classifications. Of the 501 patients enrolled, 220 (43.9%) patients had no coronary collaterals. In subjects with collaterals, the coronary grade was distributed as follows: 91(18.2%) with grade 1, 133 (26.5%) with grade 2 and 57 (11.4%) with grade 3. Table 1 compares the baseline characteristics between the poor group (n = 311) and the good group (n =190). The patients in the poor group had a trend to have lower cholesterol level (199.7 ± 45.1 vs 208.3 ± 53.6, p = 0.06), lower low-density lipoprotein cholesterol (LDL-C) level (128.7 ± 38.3 vs 136.2 ± 43.5, p = 0.066), less angiotensin II receptor blocker (ARB) usage (15.9% vs 22.3%, p = 0.09), and less beta blocker usage (28% vs 36.7%, p = 0.054).

**Table 1:** Baseline characteristics in patients with poor and good collaterals

**Variables**	**Poor collateral**	**Good collateral**	**P value**
	**(n = 311)**	**(n = 190)**	
Age (years)	62.8 ± 12.9	62.1 ± 12.1	0.547
Gender (Female, %)	22.5	18.4	0.309
DM (%)	42.8	40.5	0.642
HTN (%)	64.3	65.3	0.848
Smoking (%)	60.5	58.2	0.639
BMI	24.3 ± 6.6	24.9 ± 5.7	0.277
eGFR (mL/min/1.73 m2)	66.9 ± 26.9	68.0 ± 32.0	0.683
**Laboratory data**			
Glucose (mg/dl)	137.7 ± 62.2	135.4 ± 60.4	0.704
Cholesterol (mg/dl)	199.7 ± 45.1	208.3 ± 53.6	0.060
LDL-C (mg/dl)	128.7 ± 38.3	136.2 ± 43.5	0.066
HDL-C (mg/dl)	37.4 ± 9.9	36.0 ± 10.6	0.175
Triglyceride (mg/dl)	168.9 ± 124.9	163.4 ± 146.4	0.660
Uric acid (mg/dl)	6.54 ± 1.94	6.58 ± 1.81	0.819
**Medication**			
ACEI (%)	19.1	20.7	0.725
ARB (%)	15.9	22.3	0.09
Beta blocker (%)	28	36.7	0.054

ACEI: angiotensin converting enzyme inhibitor; ARB: angiotensin II receptor blocker; BMI: body mass index; DM: diabetes; HTN: hypertension; eGFR: estimated glomerular filtration rate; HDL-C: high-density lipoprotein cholesterol; LDL-C: low-density lipoprotein cholesterol.

Angiographic characteristics in patients with poor and good collaterals are shown in Table 2; the patients with poor collaterals had fewer diseased vessels numbers (1.97 ± 0.84 vs 2.47 ± 0.68, p < 0.001), and lower severity scores of CAD (2.65 ± 1.63 vs 3.76 ± 1.78, p < 0.001).

**Table 2:** Angiographic characteristics in patients with poor and good collaterals

**Mean±SD**	**Poor collateral**	**Good collateral**	**P value**
**Number (%)**	**(n = 311)**	**(n = 190)**	
Number of diseased vessels	1.97 ± 0.84	2.47 ± 0.68	<0.001
Significant CAD, n (%)			
1 vessel disease (%)	114 (36.7)	20 (10.5)	
2 vessel disease (%)	93 (29.9)	60 (31.6)	<0.001
3 vessel disease (%)	104 (33.4)	110 (57.9)	
Diffuse score of CAD	2.65 ± 1.63	3.76 ± 1.78	<0.001

CAD: coronary artery disease.

### Binary logistic regression analysis

Age, sex, significant variable in the univariate analysis and relevant variables including cholesterol level, LDL-C level, HDL-C level, ARB usage, and beta blocker usage were included in the multivariate logistic regression analysis. We found the number of diseased vessels (OR = 0.410, 95% CI = 0.31-0.55, p < 0.001) was the only significantly independent predictor of poor coronary collaterals development (Table 3).

**Table 3:** Multivariate logistic regression analysis of collateral circulation (good collateral group as reference group)

	**B**	**OR**	**95% CI**	**p value**
Gender (male vs female)	-0.268	0.765	0.42-1.41	0.390
Age	0.010	1.010	0.99-1.03	0.326
Cholesterol (mg/dl)	0.003	1.003	0.99-1.01	0.440
LDL-C (mg/dl)	-0.007	0.993	0.98-1.00	0.135
HDL-C (mg/dl)	0.018	1.018	0.99-1.04	0.160
ARB usage	-0.422	0.656	0.37-1.17	0.155
Beta blocker usage	-0.380	0.684	0.42-1.11	0.126
Number of diseased vessels	-0.892	0.410	0.31-0.55	<0.001

ARB: angiotensin II receptor blocker; HDL-C: high-density lipoprotein cholesterol; LDL-C: low-density lipoprotein cholesterol.

## Discussion

In the current study, we investigated the influence of HDL-C on coronary collateral formation in Chinese population with SCAD and found two major findings. First, HDL-C was not significantly associated with coronary collateral formation in a Chinese population as a previous study in a Turkish population. Second, the number of diseased vessels was the only significantly independent predictor of poor coronary collaterals in the Chinese population. This finding was also different from previous studies in a Turkish population.

### The association between HDL-C level and coronary collaterals

HDL-C has been reported to have anti-atherogenic, anti-inflammatory, and antioxidant properties in the literature [14-16]. Although Sumi M et al. also showed that reconstituted HDL-C directly stimulates endothelial progenitor cell differentiation via phosphatidylinositol 3-kinase/Akt pathway and enhances ischemia-induced angiogenesis, we did not find HDL-C was associated with coronary collateral development in this population study [17]. In addition, Low HDL-C level was a strong risk factor for coronary artery disease and was associated with poor cardiovascular outcome [1-3]. In a previous study, Turhan H et al. reported that the development of coronary collateral vessels is poorer in patients with metabolic syndrome with advanced ischemic heart disease than in control participants without metabolic syndrome in a Turkish population, which suggested that metabolic syndrome is one of the significant factors affecting the development of coronary collateral vessels adversely [18]. Although low HDL-C is one of the diagnostic criteria for metabolic syndrome, they did not report and we also did not find HDL-C was associated with coronary collateral formation. Recently, Kadi H et al. further investigated the relationship between HDL-C and coronary collateral formation in patients with SCAD and found that there was a positive relationship between HDL-C and coronary collateral formation and suggested that low HDL-C is an independent predictor of poor coronary collateral formation in a Turkish population [10]. However, Li CC et al. reported that severity of coronary stenosis was the major influencing factor in coronary collateral formation and function in a Chinese population. Serum HDL-C was not associated with coronary collateral formation, but related to coronary collateral function which was evaluated by the size of the collateral connection diameter [19]. In our study, we analyzed 501 Chinese patients with SCAD to evaluate the correlation between HDL-C and coronary collaterals. Our result showed there was no significant association between HDL-C and coronary collateral formation not only in univariate but also in multivariate analysis, which was different from a previous study in a Turkish population. The advantages of our study were that we had a larger sample size and selected more severe coronary artery lesions than the previous study [10]. In their study, the sample size was relatively small and vessels with 50-70% stenosis were selected for coronary collateral evaluation, which may not cause severe myoischemia to induce coronary collateral formation [6]. In addition, the influence of HDL-C for coronary collateral development might not be similar between different races because of genetic diversity. However, the detailed mechanism still needs further investigation.

### The association between CAD severity and coronary collaterals

It is well known that coronary collateral formation is mainly dependent on CAD severity [20-22]. Coronary collateral formation is an adaptive response to chronic myoischemia and serves as a conduit bridging significantly stenotic coronary vessels. More severe and extensive myocardial ischemia such as multi-vessel diseases may further stimulate the development of coronary collateral circulation. Hence, patients with good coronary collaterals appear to have a more extensive CAD. In previous studies, number of diseased vessels was a significant predictor of good collateral formation [20,23]. In our study, number of diseased vessels was not only associated with good collateral formation in univariate analysis but in multivariate analysis as well. In addition, we also used DS to further evaluate the extent of CAD severity and found that there was also a significant difference of DS between patients with poor and good coronary collaterals. Hence, extent of coronary atherosclerosis significantly plays an important role in coronary collateral formation [24,25].

### Limitations of the present study

First, the collateral formation was assessed by coronary angiography in this study. Measuring collateral flow index by intravascular Doppler guidewire may provide a more objective physiological measurement of collateral grade. However, the invasiveness of intravascular ultrasound limits its use in large-scale studies. Second, since this was only a clinical observational study, potential mechanisms were not fully elucidated. Third, we did not analyze the size of the collateral connection diameter to evaluate the coronary collateral function.

## Conclusions

In conclusion, our data revealed that HDL-C was not associated with coronary collateral formation in a Chinese population with SCAD; however, CAD severity implicated by number of diseased vessels was the only significant predictor of coronary collateral development in our study.

## Competing interests

The authors have no competing interests to declare.

## Authors’ contributions

PCH: study concept and design, analysis and interpretation of data and drafting the manuscript. HMS, SHJ, SHS, and WTL: study concept and design, acquisition of subjects. HWY, WCV: Providing intellectual content of critical importance to the work described. THL: study concept and design, preparation of manuscript, and final approval of the version. All authors read and approved the final manuscript.
